# Salvage Brachytherapy for Castration-Resistant and External Beam Radiotherapy-Resistant Local Recurrence 17 Years after Radical Prostatectomy

**DOI:** 10.1155/2015/839738

**Published:** 2015-06-11

**Authors:** Shogo Hosogoe, Osamu Soma, Teppei Matsumoto, Atsushi Imai, Shingo Hatakeyama, Takahiro Yoneyama, Yasuhiro Hashimoto, Takuya Koie, Chikara Ohyama, Masahiko Aoki

**Affiliations:** ^1^Department of Urology, Hirosaki University Graduate School of Medicine, 5 Zaifucho, Hirosaki 036-8562, Japan; ^2^Department of Radiology, Hirosaki University Graduate School of Medicine, 5 Zaifucho, Hirosaki 036-8562, Japan

## Abstract

A 47-year-old Japanese man was diagnosed with prostate cancer in February 1995 (Initial PSA 77.2 ng/mL, GS3 + 4, cT3N0M0). He underwent radical prostatectomy after androgen deprivation therapy (ADT) in June 1995. Nine years after operation, he was diagnosed with local recurrence of prostate cancer and he received postoperative external beam radiation therapy (EBRT) (70 Gy). By May 2008, the PSA dropped to 0.33 ng/mL, and a CT scan showed that the mass had disappeared. On April 2012, the PSA once again rose to 3.1 ng/mL. CT scan and MRI revealed a mass in the prostatic bed. We diagnosed local recurrence of prostate cancer. We underwent salvage low-dose brachytherapy after obtaining informed consent. The prescribed dose of the salvage brachytherapy was 145 Gy to control the tumor considering the hormone resistant prostatic cancer and high-risk feature. PSA level rapidly decreased to 0.66 ng/mL by 6 months after seed implantation. No adverse events were seen during the follow-up period.

## 1. Background

There are various methods of treatment for local recurrence after radical prostatectomy for prostate cancer. We hereby report the case of a patient who underwent low dose rate brachytherapy for castration-resistant and external beam radiotherapy-resistant local recurrence of prostate cancer after prostatectomy.

## 2. Case Report

**Figure 1:**
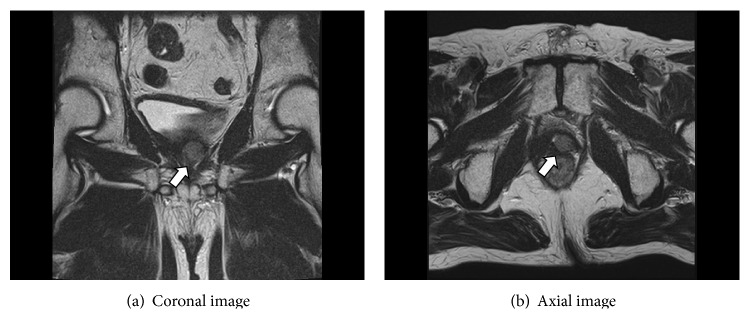
MRI images of local recurrence. T2 weighted image demonstrated a low-intensity area in the left side of prostatic bed.

A 47-year-old Japanese man was diagnosed with prostate cancer in February 1995 (Initial PSA 77.2 ng/mL, GS3 + 4, cT3N0M0). He underwent radical prostatectomy after androgen deprivation therapy (ADT) at the Aomori City Hospital in June 1995. In August 2000, the prostate-specific antigen (PSA) rose to 14.7 ng/mL, and leuprorelin hormone therapy was initiated (nadir PSA: 0.5 ng/mL). In January 2004, the PSA rose to 6.1 ng/mL and therefore bicalutamide was also administered. However, the PSA rose to 12.3 ng/mL in October 2006, and a computed tomography (CT) scan revealed a mass in the prostatic bed. A biopsy from this site confirmed the recurrence of prostate cancer; thus, the patient was subjected to postoperative external beam radiation therapy (EBRT) (60 Gy). By May 2008, the PSA dropped to 0.33 ng/mL, and a CT scan showed that the mass had disappeared. Therefore, the administration of leuprorelin and bicalutamide was discontinued. On April 2012, the PSA once again rose to 3.1 ng/mL; therefore, leuplin and estramustine were administered. CT scan and MRI revealed a mass in the prostatic bed (Figure 1). There was no evidence of distant metastasis or lymph node metastasis. We diagnosed local recurrence of prostate cancer. We decided to undergo salvage brachytherapy after informed consent.

**Figure 2:**
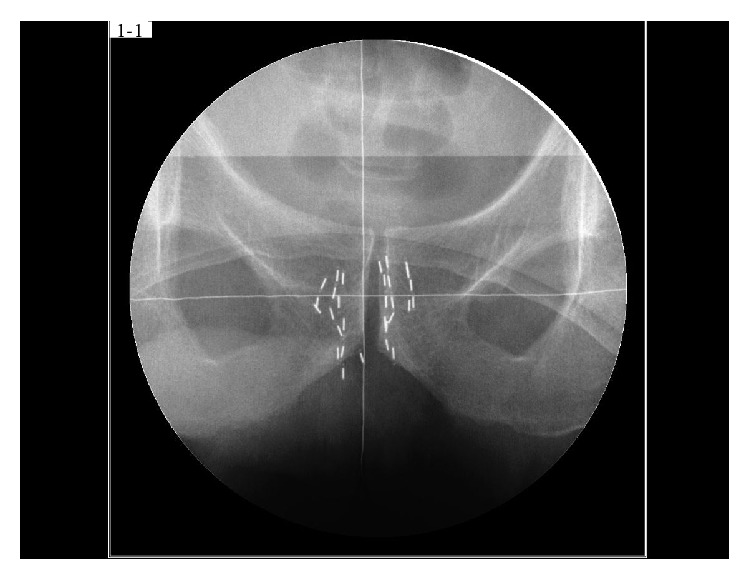
Pelvic X-ray image after salvage brachytherapy.

Prior to salvage brachytherapy, biopsy was performed and the histological findings showed an adenocarcinoma with a Gleason score of 5 + 4. Although external beam radiotherapy of 70 Gy has been delivered prior to salvage brachytherapy, the prescribed dose of the salvage brachytherapy was 145 Gy to control the tumor considering the hormone-resistant prostatic cancer and high-risk feature. The tumor volume using transrectal ultrasound (TRUS) was 5.9 mL at a preplanning. We used a radioactive source of 0.27 mCi (11.0 MBq) according to tumor size as same as standard brachytherapy. Thirteen needles were inserted into the tumor based on a peripheral loading technique, and 2 or 3 sources were implanted through each needle. Finally 30 sources were implanted by the real-time planning method (Figure 2). Regarding dosimetric parameters using CT image 1 month after salvage brachytherapy, tumor D90 was 178 Gy, tumor V100 was 98%, the rectal V100 was 0.01 mL, and the minimal dose received by 30% of the urethra (UD30) was 230 Gy. The treatment time was 100 minutes. Since the PSA level rapidly decreased to 0.66 ng/mL by 6 months after seed implantation, he received no adjuvant therapy. 18 months after seed implantation, the PSA level reached 1.12 ng/mL. No adverse events were seen during the follow-up period.

## 3. Discussion

When the patients show PSA recurrence or local recurrence after radical prostatectomy, there are several salvage treatments such as ADT, radiation therapy, chemotherapy, and surgical therapy [1–9].

Our case exhibited local recurrence 11 years after undergoing radical prostatectomy and he underwent EBRT. He made a complete response; however, 6 years after EBRT, local recurrence was occurred again; therefore, low dose rate (LDR) brachytherapy was performed.

MacDonald et al. investigated 102 patients who underwent EBRT for isolated PSA elevation or palpable local recurrence after radical prostatectomy [1]. They reported that the 5-year rate of biochemical disease-free survival (bDFS), local control, freedom from distant metastases, and overall survival for all 102 men were 38%, 94%, 87%, and 88%, respectively. They also reported that five patients (5%) experienced chronic grade 3 or 4 radiotherapy-related toxicity. Traudt et al. performed LDR brachytherapy on five patients with local recurrence after radical prostatectomy and found that it offered good PSA control while the genitourinary and sexual side effects were minor and infrequent, and no rectal toxicity occurred [2].

Because our patient exhibited post-EBRT local recurrence, we concerned radiation related complication after LDR brachytherapy. Lee et al. investigated 21 cases of post-EBRT salvage brachytherapy and only noted PSA failure in four patients and distant metastasis in one patient 59 months after treatment, with no adverse events of grade 3 or higher [3]. Our patient also exhibited good PSA control after LDR brachytherapy without any complications.

In conclusion, we reported a case of salvage brachytherapy for castration-resistant and external beam radiotherapy-resistant local recurrence 17 years after radical prostatectomy. Longer follow-up data are needed to establish the usefulness of salvage brachytherapy for castration-resistant and external beam radiotherapy-resistant local recurrence after radical prostatectomy.
